# Selection on Crop-Derived Traits and QTL in Sunflower (*Helianthus annuus*) Crop-Wild Hybrids under Water Stress

**DOI:** 10.1371/journal.pone.0102717

**Published:** 2014-07-21

**Authors:** Birkin R. Owart, Jonathan Corbi, John M. Burke, Jennifer M. Dechaine

**Affiliations:** 1 Department of Biological Sciences, Central Washington University, Ellensburg, Washington, United States of America; 2 Department of Plant Biology, University of Georgia, Athens, Georgia, United States of America; University of Arkansas, United States of America

## Abstract

Locally relevant conditions, such as water stress in irrigated agricultural regions, should be considered when assessing the risk of crop allele introgression into wild populations following hybridization. Although research in cultivars has suggested that domestication traits may reduce fecundity under water stress as compared to wild-like phenotypes, this has not been investigated in crop-wild hybrids. In this study, we examine phenotypic selection acting on, as well as the genetic architecture of vegetative, reproductive, and physiological characteristics in an experimental population of sunflower crop-wild hybrids grown under wild-like low water conditions. Crop-derived petiole length and head diameter were favored in low and control water environments. The direction of selection differed between environments for leaf size and leaf pressure potential. Interestingly, the additive effect of the crop-derived allele was in the direction favored by selection for approximately half the QTL detected in the low water environment. Selection favoring crop-derived traits and alleles in the low water environment suggests that a subset of these alleles would be likely to spread into wild populations under water stress. Furthermore, differences in selection between environments support the view that risk assessments should be conducted under multiple locally relevant conditions.

## Introduction

Although gene flow from cultivated to wild populations has likely been occurring since the domestication of wild lineages, interest in the topic has increased with the commercialization of transgenic crops. Consequences of crop-wild hybridization may include the escape of engineered genes into wild populations [1], increased invasiveness of wild relatives of the cultivar [2] [3], and the potential for crop-wild hybrids to outcompete native taxa [4]. Crop-wild hybridization must thus be considered when discussing the potential impacts of transgenic cultivars. The selective advantage of an allele is the best predictor of its establishment and spread in a new population [5] [6], and potentially advantageous transgenic alleles have been documented in *Cucurbita pepo* [7], *Helianthus annuus* [8], and *Oyrza sativa* [9]. These studies suggest that transgenes can increase fitness in crop-wild individuals following introgression, but the ubiquity of their effects on wild populations under diverse selective environments is largely unknown.

Although much attention has been given to the escape of engineered transgenes, crop-wild hybridization may also contribute advantageous natural alleles to wild populations and can thus be used to model how selection acts on potential targets for genetic engineering. Hybridization between non-transgenic cultivars and wild relatives has resulted in the generation of at least seven types of agricultural weeds [10], and range expansion of wild populations following crop-derived allele introgression has been observed in *Sorghum halapense*, *Rhododendron ponticum*, and *Manihot reptans* [11]. Studies in sunflower have suggested that crop-like traits (i.e., earlier flowering time and a larger primary inflorescence) increase reproductive output in crop-wild hybrids under a range of natural conditions [12] [13]. Crop-derived alleles have been known to persist within wild populations for at least five generations following hybridization in sunflower [14] and ten generations in wild radish [15], suggesting that some crop-derived alleles may persist in the wild, contrary to expectation. Indeed, crop-derived alleles may have also contributed to the evolution of weediness in wild sunflower [16]. Estimations of selection for crop-derived traits and alleles in wild environments may be applied toward understanding the consequences of transgene escape into the wild.

The fitness effects of a trait or allele likely differ with environmental factors. For example, when exposed to increased interspecific competition and herbicide application, the relative fitness of sunflower crop-wild hybrids increased compared to their wild counterparts, suggesting that crop-like traits are more advantageous under certain conditions [17] [18]. Studies in sunflower also show that crop-like flowering (early) was advantageous in the absence of herbivory [12] [13], but wild-like flowering (later) was favored when pre-dispersal herbivory was considered [13]. Increased pre- and post-dispersal predation has been observed for F_1_ crop-wild hybrids relative to wild sunflower individuals [19] [20], suggesting that the presence of such herbivores could reduce hybrid fitness. The spread of otherwise advantageous crop-derived alleles may thus be mitigated by natural environmental factors. As such, fitness should be assessed over a range of locally relevant conditions.

Water stress is an important environmental factor influencing the fitness of crop-derived traits and alleles that has not been studied in crop-wild hybrids. Under drought conditions, plants may be more susceptible to disease and consequently derive greater benefit from transgenic disease resistance [21]. Given that there will be heightened demand for transgenic drought tolerance technologies since irrigated agriculture will increasingly be operating under water scarcity [22], it is important for crop-wild hybridization risk assessments to identify crop-like traits conveying success under water stress in natural habitats.

Characteristics that result in the efficient utilization of leaf surface area and corresponding leaf water potential at turgor loss are likely to be advantageous under arid conditions [23]. Cultivated sunflowers typically do not display these characteristics as selection for growth in water abundant agricultural environments has resulted in a trade-off with drought tolerance [24]. In cultivated sunflower lineages, water stress reduces stem height, stem diameter, number of leaves, and leaf area [25] [26]. Decreased shoot and inflorescence mass have been observed in both cultivated and wild sunflowers when exposed to water stress, but cultivated individuals were more susceptible to drought in terms of wilting morphology [27]. Nevertheless, it is possible that a subset of crop-derived traits and alleles may confer an advantage in arid environments following introgression into wild populations.

The application of quantitative trait locus (QTL) mapping to crop-wild systems can elucidate the fitness effects of cultivar-derived alleles in natural environments. Estimates of crop-derived allele additive effects among loci from QTL mapping, coupled with phenotypic selection analyses, improve our ability to predict if crop-derived alleles will introgress into wild populations. Crop-derived alleles with strong additive effects that affect traits conferring a fitness advantage under diverse wild-like environments have a high probability for introgression. Comparisons of significant QTL may be made across populations and environments, allowing for the identification of crop-derived alleles that are likely to be favored across a wide range of relevant natural conditions [28].

Here we assess selection on crop-like traits in sunflower crop-wild hybrids exposed to water stress. We then infer patterns of selection on QTL by predicting how selection may affect crop-derived alleles in the tested environments. Specifically, we use a low water treatment in comparison to a control water treatment to investigate: (i) the direction and magnitude of selection acting on crop-like traits in each environment; (ii) the genetic architecture of these characteristics; and (iii) the inferred fitness effects of crop-derived alleles under these conditions.

## Materials and Methods

### Study System

Common sunflower (*H. annuus* L.) is one of five highest production oilseed cultigens worldwide [29] and a model for studying crop-wild gene flow. Its weedy, self-incompatible wild relative (*H. annuus* var. *annuus*) is native to North America and has a rangeF extending throughout that of cultivated sunflower [30] [31] with abundance in the central and western United States [32]. Centuries of artificial selection have resulted in morphological divergence. Differences between cultivated and wild sunflower are readily observed in the number and size of heads, branching, leaf shape and size, and seed (achene) size [33]. Cultivated and wild sunflower populations frequently hybridize [34] [35], and crop-derived alleles are known to persist in the wild [14].

### Mapping Population and Experimental Design

As previously described [33] [12] [36] recombinant inbred lines (RILs) were developed from a cross between an oilseed cultivar (cmsHA89, PI650572) and a wild *H. annuus* var. *annuus* individual (Ann1238, PI659440) from Keith County, NE, USA. Seed for the present study came from the F_7_–F_9_ generations of 146 RILs from this population.

Between 21–24 May 2012, eight seeds per experimental entry (RILs and parents) were germinated in a greenhouse at Central Washington University (CWU), Ellensburg, WA before transplanting into the field. Seeds of all lines were either sown directly into biodegradable pots (Jiffy, 3″x3″) with field-collected soil at a depth of 2.5 cm (91 RILs) or, in the case of lines with known poor or undetermined germination rates, subjected to a dormancy-breaking treatment prior to planting (55 RILs). The soil type was a mitta ashy silt loam with a high available water capacity [37]. The dormancy-breaking treatment involved starting seeds that had been sterilized with 3% hydrogen peroxide on filter paper in Petri dishes, treating them with 25 ppm Ethephon for 24 hours, and transplanting them into the greenhouse upon cotyledon emergence.

Upon emergence of the first true leaves, 18–28 June 2012, individuals of the 58 RILs with at least four replicates per line were transplanted into the field at CWU using a randomized complete block design. Replicates of each RIL were evenly split among four blocks and fully randomized across 24 paired rows within each block. Spacing was 0.3 m between paired rows and 0.4 m between plants within a row. The field was tilled before transplanting to simulate disturbed habitat that wild sunflower populations frequently colonize. The field site was located from the nearest wild sunflower population by ca. 40 km, thereby ensuring isolation. This region, which is an important irrigated agricultural area, receives less than 5 cm of precipitation and has daytime relative humidity of ca. 45% during the June – September period when this experiment took place.

To help ensure establishment, seedlings were watered twice weekly before beginning water treatments on 24 July 2012. At that time, two blocks each were assigned to control water (CW) and low water (LW) treatments, to simulate regional cultivated and wild sunflower water conditions, respectively. CW blocks were spray irrigated for two hours once a week, and LW blocks were not watered throughout the growing season. Irrigation ceased in the LW blocks pre-flowering to stress plants during reproductive growth phases. Hand weeding was performed to minimize the effect of asymmetric interspecific competition between treatments.

### Plant Characteristics

Plants began flowering on 1 August 2012. Flowering day was recorded upon opening of the first disc floret on the primary inflorescence of the first flowering plant. At peak flowering, plant water status was collectively evaluated for each individual using leaf pressure potential and leaf water content. The largest leaf of each plant was collected pre-dawn on 9 September 2012 and used to measure leaf pressure potential in the laboratory using a standard pressure chamber (Soilmoisture Equipment Corp.). Leaf water content was measured on the same leaf as a proportion of dry to wet mass; each leaf was massed before and after drying for three days in an oven at 32°C.

At the start of senescence (sunflower developmental stage, R-8) [38], approximately 15 weeks of age, morphological characteristics were measured. Stem diameter was measured at the base of the individual and plant height was measured from the base to the capitulum along the primary stem. Petiole length, leaf (blade) length, and leaf width were recorded for the largest leaf on the plant. Head diameter was determined for the primary inflorescence. Head total refers to the number of developing capitula exceeding 3 cm in diameter. Branch number was counted as the number of axillary shoots developed from the primary shoot. All seeds were collected and weighed for each individual. Total seed mass and the mean mass of ten seeds were used to indirectly estimate total fecundity (i.e., total number of seeds produced). Leaf size was estimated by multiplying leaf length by width.

### Quantitative Genetic and Selection Analysis

Only RILs with data from at least two replicates in each water treatment for all characteristics were included in analyses (except for the selection analyses), resulting in a total of 237 individuals from 32 RILs (see discussion). All analyses were conducted using R [39], except for QTL mapping. A restricted maximum likelihood (REML) approach was used to test for the fixed effects of treatment, block, and planting date, as well as the random effects of line (RIL) and line-by-treatment on each characteristic (lme4 package) [40]. Models including only one random effect were compared separately (RLRsim package) [41]. Mixed model F-statistic significance scores were also produced (lmerTest package) [42]. All characteristics met model assumptions. Models were then used to calculate least-squares means (lsmeans package) [43] and variance components; broad-sense heritability was considered as a proportion of additive genetic (RIL) to total variance. REML models generated best linear unbiased predictors (BLUPs) used to estimate bivariate genotypic Pearson correlations within each treatment.

Aster models for life-history traits were used to characterize phenotypic selection on characteristics within each water treatment (aster package) [44]. These models utilize maximum likelihood linear methods and improve upon previous least squares methods by modeling multiple components of fitness into a single variable, as well as by specifying a particular distribution for each component of fitness [45] [46] [47]. All characteristics were regressed with the combined fitness variable based on two components: survival to reproduction (Bernoulli distribution) and fecundity (truncated Poisson distribution). In order to account for selection on plants that died prior to reproduction, all individuals that survived to the initiation of watering treatments were included in selection analyses. Final sample sizes were 476 individuals from 62 RILs in the CW treatment and 67 RILs in the LW treatment, respectively. Relative fitness was calculated as a proportion of mean population fecundity in each treatment [48]. Block was included as a model parameter. To test for significant effects of each trait, sub-models, each omitting one characteristic (df = 12, each), were compared against the full model (df = 13) using likelihood ratio tests. We also used aster to calculate 95% selection gradient (β) confidence intervals for each trait [49]. Variance inflation factors (VIF) [50] were used to assess whether any two characteristics violated assumptions of multicollinearity; however, no combination achieved a VIF greater than five and all characteristics were thus retained in the selection model.

Crop- or wild-like designations were assigned to either positive or negative linear selection for vegetative and reproductive growth characteristics using previous trait assignments for these RILs [33] [13] and by comparing the mean cultivated parent trait value to the RILs. For example, when irrigated, the cultivar developed a much larger head diameter than the RIL population (Table S1), supporting that a larger head is a crop-like trait. Few seeds of the wild parental population germinated, likely due to high dormancy, and data for it could not be obtained. Leaf pressure potential and water content could not be assigned a crop- or wild-like distinction because these characteristics were strongly influenced by water treatment (Table 1; see discussion).

**Table 1:** Genetic and environmental effects on morphological and physiological characters.

	Fecundity	Stem Diameter	Plant Height	Petiole Length	Leaf Size	Branch Number	Head Diameter	Head Total	Days to Flower	Leaf PP	Water Content
**Random Effects**											
**RIL**	9.32*	39.88***	74.23***	22.29***	35.12***	40.81***	22.48***	28.24***	111.42***	9.68***	1.42
RIL*Treatment	2.87	26.01***	24.07***	9.39***	59.72***	29.82***	0.20	33.30***	2.38	0.00	0.00
**Fixed Effects**											
**Block (Treatment)**	5.77**	6.47**	6.39**	9.45**	0.16	1.57	4.27*	8.77*	5.05**	0.58	1.47
**Treatment**	36.48***	17.82***	19.24***	13.88**	12.59**	25.16***	23.85***	12.85**	9.67**	68.34***	0.12
**Planting Date**	1.39	2.35	2.03	1.01	2.04	1.05	4.13	0.76	46.12***	0.31	0.87

Fixed and random effects for morphological and physiological characters measured in recombinant inbred line (RIL) sunflower cultivar (cmsHA89) x wild (ann1238) hybrids. Chi-square (random) and F-statistic (fixed) values were extracted from a restricted maximum likelihood model: *, *P*<0.05; **, *P*<0.01; ***, *P*<0.001.

### Genetic Mapping

Total genomic DNA of the full set of 169 RILs was isolated from 200–400 mg fresh leaf tissue (142 RILs) using the DNeasy Plant Maxi Kit (Qiagen). For the 27 RILs that could not germinate, gDNA was directly extracted from 100 mg of seeds. Samples were initially ground in 2 mL tubes using a TissueLyser (Qiagen) before 1 mL of washing buffer was added to each sample [51]. Tubes were mixed on ice for 5 min and then centrifuged at 16,000 rpm for 10 min at 4°C. The supernatant was discarded and the pellet was used to extract gDNA following the DNeasy protocol provided by Qiagen. The quality of the DNA was evaluated by spectrophotometry using a NanoDrop ND-1000 (Thermo Fisher Scientifc). To ensure that the quality of the DNA extracted from seeds and leaf led to equivalent results, 23 randomly chosen RILs were genotyped using both types of tissues; no differences were observed between tissue types.

An array targeting 384 single nucleotide polymorphisms (SNP) was developed from a subset of the SNPs present on the 10,640 feature Illumina Infinium array described by Bachlava et al. [52]. SNPs were selected based on known polymorphism between the parents based on an initial screen with the larger array as well as known map positions [53]. Genotyping was based on the Illumina GoldenGate platform and the VeraCode technology (Illumina, San Diego, CA). Genotypes were manually called using GenomeStudio V2011.1 (Illumina). A total of 353 SNPs were successfully scored and thus included in the analysis. Correlations between SNPs were estimated using the Pearson coefficient using the *cor* function [54] in R [39]. Visual examination of the correlation matrix as a heat map revealed that four SNPs (SFW00635, SFW08150, SFW00608, SFW05812) exhibited strong associations with distant positions in the genome. Given the history of duplication in the sunflower genome [55], this was presumably due to the mapping of different paralogs in different populations, as has been previously documented in sunflower [53]. Consequently, the relative positions of all SNPs for the 17 linkage groups were re-calculated using MAPMAKER/EXP 3.0 [56] [57] knowing *a priori* the assignment to each linkage group (LG). The Kosambi distance function was set for all linkage groups. The order of markers presumably belonging to a same LG was initially inspected using the “LOD” command. Based on this pairwise comparison matrix providing the distance (in cM) between SNPs and LOD scores, we made a core group of markers less than 5 cM apart and the relative order was evaluated by the “compare” command. The most likely order was kept and the remaining SNPs to be incorporated to the LG were sequentially added using the “try” command. At every step, the new order was evaluated using the “ripple” command. Once all SNPs were assigned to a LG, the map was enriched by adding 140 previously mapped simple sequence repeat (SSR) markers [58]. Each SSR was initially assigned to a LG using the “near” command. The relative position of the SSR within a LG was estimated using the “try” command and the final order was then checked using the “ripple” command.

### QTL Mapping

QTL mapping was conducted using the SNP/SSR map on BLUPs for all characteristics using a composite interval mapping (CIM) protocol in QTL Cartographer [59]. Forward and backward regression settings were chosen (*P* = 0.05) with a walk speed of 2 cM. Significant logarithm of odds thresholds (LOD) were estimated with 1000 permutations tests per characteristic [60]. QTL Cartographer was used to calculate the additive effect of the cultivar allele and the percent variance explained by each QTL. The additive effect was standardized to one standard deviation. Multi-trait mapping to assess QTL × environment interactions was conducted in QTL Cartographer using aforementioned CIM settings [61].

## Results

### Treatment Effects

Significant genetic variation was observed (RIL effects) for all characteristics except water content and RILs differed in their response to the water treatments (RIL × treatment effects) for stem diameter, plant height, petiole length, leaf size, branch total, and head total (Table 1). For all characteristics, the degree of heritability was lower in the LW than the CW treatment (Table 2). Of note, the heritability of leaf pressure potential and water content in the LW treatment approached 0.00, suggesting little additive genetic control for these characteristics.

**Table 2:** Character means and heritabilities.

	Mean (SD)		Heritability (SE)		Range (low, high)	
	CW	LW	CW	LW	CW	LW
**Fecundity**	365.56 (421.80)	107.83 (127.10)	0.24 (1.17E-4)	7.33E-10 (2.15E-8)	(0.33, 1238.00)	(4.00, 309.80)
**Stem Diameter (mm)**	7.95 (4.74)	5.34 (2.11)	0.41 (9.02E-3)	0.11 (9.55E-3)	(2.84, 24.24)	(2.57, 8.57)
**Plant Height (cm)**	56.98 (23.81)	42.93 (15.30)	0.64 (2.78E-3)	0.37 (3.78E-3)	(25.05, 126.83)	(21.30, 70.24)
**Petiole Length (cm)**	5.19 (3.83)	3.53 (1.97)	0.24 (9.21E-3)	2.84E-10 (7.29E-7)	(1.20, 14.28)	(6.75, 88.20)
**Leaf Size (cm^2^)**	82.13 (4.09)	34.35 (31.21)	0.73 (7.48E-4)	7.81E-10 (8.86E-8)	(3.80, 20.87)	(6.75, 88.20)
**Branch Number**	5.35 (4.59)	2.22 (2.04)	0.40 (1.04E-2)	0.21 (2.38E-2)	(0.33, 15.80)	(0.00, 5.00)
**Head Diameter (mm)**	44.84 (21.05)	37.56 (12.63)	0.21 (2.48E-3)	0.16 (2.88E-3)	(13.88, 119.10)	(18.50, 61.80)
**Head Total**	10.27 (9.04)	5.28 (2.84)	0.44 (5.64E-3)	0.08 (6.76E-3)	(2.40, 33.00)	(1.50, 10.00)
**Days to Flower**	76.38 (12.72)	77.29 (11.99)	0.62 (6.38E-3)	0.44 (5.41E-3)	(67.00, 112.40)	(69.25, 111.00)
**Leaf Pressure Potential (MPa)**	3.27 (1.37)	4.41 (0.90)	0.09 (1.16E-2)	2.29E-11 (1.86E-7)	(1.55, 6.55)	(2.85, 6.00)
**Water Content**	20.91 (10.72)	21.56 (7.55)	1.70E-10 (1.06E-7)	2.14E-10 (1.69E-7)	(13.60, 34.30)	(15.82, 34.40)

Summary statistics for morphological, reproductive and physiological characteristics in sunflower recombinant inbred line (RIL) cultivar (cmsHA89) x wild (ann1238) hybrid populations grown in control (CW) and low water (LW) treatments. Least-squares means, standard deviations (SD), and heritability components were calculated using a restricted maximum likelihood analysis. Broad-sense heritability is the proportion of line (RIL) to total variance.

With the exception of water content, watering treatment effects were significant (Tables 1). Individuals were generally smaller and had less reproductive output in the LW treatment, as well as displayed greater leaf pressure potential (Table 2). Despite significant treatment effects for most characteristics, the direction of significant among-trait correlations remained consistent across both watering regimes (Table S2).

### Selection Analyses

Most characteristics indicated significant gradients for directional selection in both treatments (Table 3). Many characteristics showed consistent selection across environments. In both treatments, greater fecundity (higher fitness) was associated with: smaller stem diameter and larger plant height, petiole length, and head diameter, as well as more branches and greater water content. In contrast, the direction of selection differed between treatments for leaf size and leaf pressure potential. In the CW treatment, plants had higher fecundity if they displayed smaller leaf size and lower leaf pressure potential; selection estimates were reversed for these characteristics in the LW treatment. A greater number of heads and early flowering day were favored in the CW treatment although selection estimates were non-significant for these characteristics in the LW treatment.

**Table 3:** Phenotypic selection analyses.

	CW			LW		
	β	Deviance	P-value	β	Deviance	P-value
**Stem Diameter**	(−3.08E-2, −2.72E-2)	1046.80	<2.20e-16	(−9.08E-2, −8.43E-2)	2509.10	<2.20e-16
**Plant Height**	(8.07E-4, 1.46E-3)	45.82	<2.20e-16	(5.53E-3, 6.63E-3)	473.78	<2.20e-16
**Petiole Length**	(5.19E-3, 8.64E-3)	66.95	<2.20e-16	(2.38E-2, 2.99E-2)	232.64	<2.20e-16
**Leaf Size**	(−6.36E-2, −4.28E-2)	110.87	<2.20e-16	(2.13E-3, 2.54E-3)	485.10	<2.20e-16
**Branch Number**	(1.40E-2, 1.77E-2)	290.06	<2.20e-16	(9.13E-2, 9.85E-2)	2674.10	<2.20e-16
**Head Diameter**	(1.14E-2, 1.18E-2)	8246.50	<2.20e-16	(3.53E-2, 3.64E-2)	17877.00	<2.20e-16
**Head Total**	(4.01E-2, 4.16E-2)	10292.00	<2.20e-16	(−1.29E-3, 3.33E-3)	0.75	0.39
**Days to Flower**	(−1.31E-3, −8.69E-4)	92.84	<2.20e-16	(−9.97E-5, 2.08E-4)	0.49	0.49
**Leaf Pressure Potential**	(−1.12E-2, −6.85E-3)	49.38	2.11e-12	(4.96E-2, 5.79E-2)	852.04	<2.20e-16
**Water Content**	(5.94E-3, 6.62E-3)	1058.00	<2.20e-16	(1.21E-3, 2.23E-3)	43.57	4.10e-11

Results summary of aster model comparisons and 95% selection gradient (β) confidence intervals for morphological, reproductive, and physiological characteristics in sunflower recombinant inbred line (RIL) cultivar (cmsHA89) x wild (ann1238) hybrids grown under control (CW) and low (LW) water treatments. Sub-models (d.f. = 12), each omitting one trait, were compared to the full model (d.f. = 13). Likelihood ratio test deviance and χ^2^ test *P*-values are shown.

Crop- or wild-like attributes may be assigned to each direction of selection for vegetative and reproductive growth characteristics. Three crop-like traits were favored by selection in each water treatment: head diameter and petiole length in both treatments, leaf size in LW and days to flower in CW. Due to strong influence of environmental variation, a crop- or wild-like attribute was not given to leaf pressure potential or water content.

### Genetic Map Construction

The genetic map was composed of the expected 17 linkage groups and included a subset of 358 of the 384 targeted SNPs and a set of 140 previously described SSR markers [58] [12] [13], resulting in a total of 498 markers. The final map covered 1968.2 cM (range  = 83.0–161.0 cM per linkage group), on par with previous studies in sunflower [62] [63], with an average intermarker interval of 3.9 cM (range  = 0.0–27.1 cM).

### QTL Mapping

**Figure 1:**
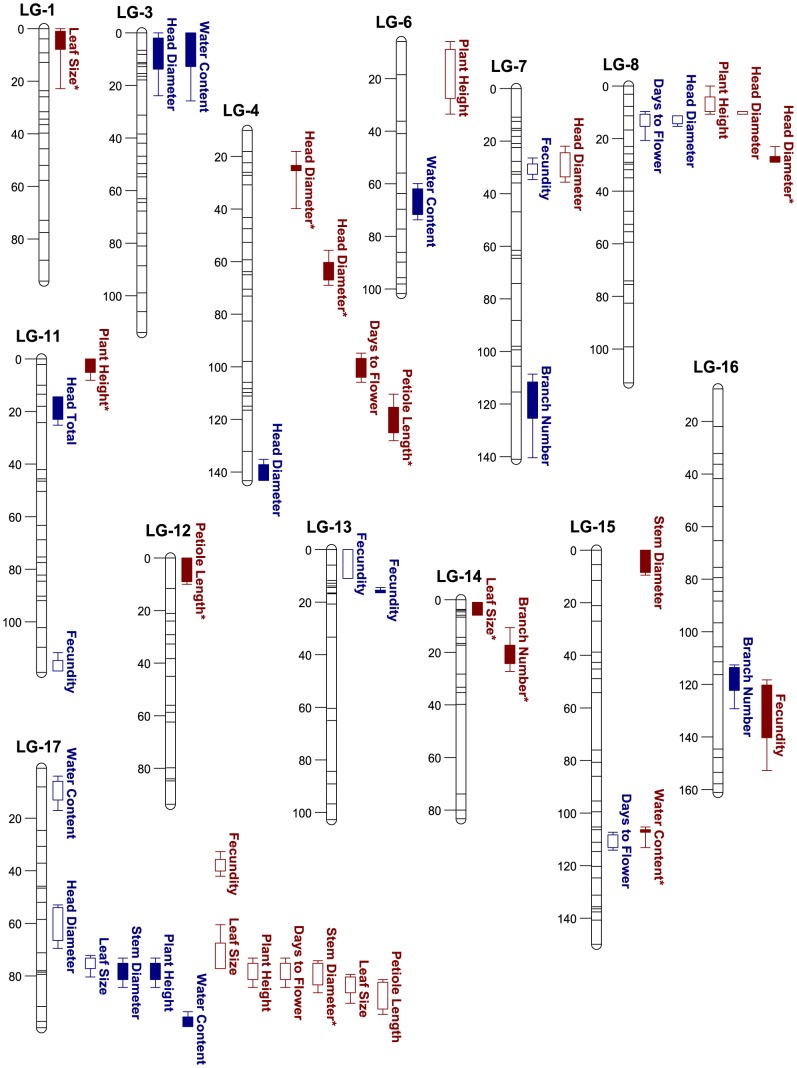
Graphical representation of quantitative trait locus (QTL) composite interval mapping results from QTL Cartographer. Characters were mapped using cultivar (cmsHA89) x wild (ann1238) sunflower recombinant inbred lines grown in control water (blue) and low water (brown) treatments. Fill indicates the positive (closed) or negative (empty) additive effect of the crop-derived allele, and asterisks mark QTL with an additive effect in the direction favored by selection. 1-LOD (box) and 2-LOD (tails) thresholds are shown.

Despite the low sample size, 44 QTL affecting ten characteristics were detected across both treatments (Table 4; Fig. 1). Twenty QTL for ten characteristics explaining 10.5–55.3% of the variance and 24 QTL for nine characteristics explaining 10.5–37.1% of the variance were mapped in the CW and LW treatments, respectively. At least one QTL was detected in both treatments for most characteristics, except plant height and leaf size in the CW treatment, head total in the LW treatment, and leaf pressure potential in both treatments. QTL × environment interactions were not statistically significant for any characteristic. Although QTL mapping in the present study utilized a small number of RILs, the number of QTL detected for traits that have been previously mapped using the same RIL population were not considerably fewer than in prior studies [12] [13] (see discussion), and 40% of these were detected on the same linkage group as previously reported for the same traits (Fig. 1).

**Table 4:** Quantitative trait loci (QTL) mapping results.

			CW			LW			
Trait	LG	Marker	2-LOD	α	PVE	2-LOD	α	PVE	Prior map
**Stem Diameter**	**17**	**q_ORS561**	**73.21–84.41**	**0.82**	**43.2**				**B02-L**
	**15**	**o_15.0657**				**0.00–9.51**	**0.52**	**20.5**	
	**17**	**q_17.3558**				**60.51–77.21**	**−0.73**	**23.2**	**B02**
**Plant Height**	**17**	**q_ORS561**	**73.21–84.41**	**0.78**	**38.2**				**B02-L**
	**6**	**f_HT135**				**5.91–33.51**	**−0.45**	**10.5**	**B02-L, B08-L**
	**10**	**j_ZVG43B**				**0.00–10.71**	**−0.35**	**10.9**	**B02-L, B08**
	**11**	**k_ORS621**				**0.00–8.11**	**0.83**	**21.8**	
	**17**	**q_ORS561**				**73.21–84.41**	**−1.31**	**29.4**	**B02-L**
**Petiole Length**	**4**	**d_4.9376**				**120.51–138.21**	**0.82**	**17.8**	**NA**
	**12**	**l_ZVG54**				**0.00–10.01**	**0.81**	**32.3**	**NA**
	**17**	**q_ORS735**				**81.41–94.61**	**−0.58**	**21.3**	**NA**
**Leaf Size**	**17**	**q_ORS561**	**72.21–80.41**	**−1.19**	**42.9**				
	**1**	**M18F17**				**0.00–22.81**	**0.74**	**20.1**	
	**14**	**n_ORS578**				**1.01–5.91**	**0.75**	**32.2**	
	**17**	**q_17.3558**				**60.51–77.21**	**−0.73**	**23.2**	
	**17**	**q_ORS735**				**79.41–90.41**	**−0.81**	**22.4**	
**Branch Number**	**7**	**g_7.5311**	**108.61–140.41**	**0.57**	**29.8**				**B02-L**
	**16**	**p_16.6437**	**112.61–129.31**	**0.77**	**18.9**				
	**14**	**n_14.1672**				**10.61–27.31**	**0.68**	**31.7**	
**Head Diameter**	**3**	**c_ORS555**	**0.00–23.91**	**0.49**	**21.8**				
	**4**	**d_HT221**	**135.21–143.21**	**0.57**	**25.4**				**B02-L**
	**10**	**j_10.1292**	**11.31–15.31**	**−0.84**	**27.3**				
	**17**	**q_ORS297**	**53.01–69.51**	**−0.61**	**26.1**				
	**4**	**d_HT298**				**18.11–39.81**	**0.51**	**16.4**	**B02-L, B08**
	**4**	**d_4.4537**				**55.71–69.01**	**0.81**	**25.1**	**B02**
	**7**	**c2588**				**21.91–35.61**	**−0.38**	**12.8**	
	**10**	**j_10.1031**				**9.71–10.71**	**−0.91**	**33.5**	
	**10**	**j_HT347**				**23.01–29.01**	**0.55**	**13.4**	
**Head Total**	**11**	**k_11.1515**	**14.41–25.21**	**0.92**	**55.3**				
**Days to Flower**	**10**	**j_10.1031**	**9.71–20.71**	**−0.67**	**28.9**				
	**15**	**o_15.4733**	**107.31–114.11**	**−0.75**	**48.1**				
	**4**	**d_4.6414**				**103.91–119.51**	**0.82**	**15.8**	**B02-L**
	**17**	**q_ORS561**				**73.21–84.41**	**−1.14**	**29.2**	**B02-L, B08-L, D09-L**
**Water Content**	**3**	**c_ORS555**	**0.00–25.91**	**1.19**	**15.4**				**NA**
	**6**	**f_6.2489**	**59.91–73.71**	**1.63**	**24.4**				**NA**
	**17**	**q_17.0175**	**4.01–17.11**	**−2.12**	**47.4**				**NA**
	**17**	**q_17.6085**	**93.61–99.31**	**1.93**	**24.9**				**NA**
	**15**	**B_1.4171**				**105.31–113.11**	**0.59**	**33.1**	**NA**
**Fecundity**	**7**	**N5M02**	**26.41–34.61**	**−0.84**	**49.8**				
	**11**	**k_HT390**	**111.71–118.71**	**−0.54**	**23.4**				
	**13**	**m_HT568**	**0.00–10.91**	**−0.69**	**30.1**				
	**13**	**m_HT568**	**14.41–16.41**	**0.69**	**29.9**				
	**16**	**p_HT52**				**118.31–152.81**	**0.54**	**25.1**	
	**17**	**q_17.2328**				**32.71–42.11**	**0.82**	**37.1**	

Quantitative trait loci (QTL) composite interval mapping results in sunflower recombinant inbred line (RIL) cultivar (cmsHA89) x wild (ann1238) hybrid populations grown under control (CW) and low (LW) water treatments. Columns 2 and 3 list the linkage group (LG) and left-flanking marker for each QTL. Columns 4–6 and 7–9 indicate the logarithm of odds (2-LOD) support thresholds in cM, the standardized additive effect (α) of the crop-derived allele (cmsHA89), and the percent variance (PVE) explained by each QTL in the CW and LW treatments, respectively. If QTL colocalize with those detected in prior studies using the same RIL population, they are coded as B02 (Burke et al. 2002b), B08 (Baack et al. 2008), and D09 (Dechaine et al. 2009); –L indicates that QTL were detected on the same LG but not overlapping and NA specifies that the characteristic was not measured in previous studies.

Similar clustering of QTL on LG 17 for seven characteristics was observed in both treatments. The additive effect of the crop-derived allele did not always confer the more crop-like trait (Table 4). For example, the crop-derived allele at two QTL (LG 7, 16) increased branch number, even though domesticated individuals have been artificially selected to display limited branching phenotypes. In addition, cultivars had lower water content on average compared to hybrid individuals in LW treatment (Table S1), despite detecting the crop-derived allele increasing water content at one QTL (LG 15) in the LW treatment. For characteristics affected by multiple QTL, only branch number and days to flower for the CW treatment, as well as fecundity in the LW treatment, indicated consistent direction of additive effects of the crop-derived allele at all QTL. Additive effects of the crop-derived allele were in the direction of phenotypic selection analysis for 64% of QTL overall, although a greater proportion of crop-derived alleles were predicted for selection in CW (75%) vs. LW (55%).

## Discussion

Whether crop- or wild-like, the majority of traits conferring a fitness advantage in both environments generally increased plant competitive ability regardless of water stress. Characteristics related to competitive ability play a particularly important role in stressful, resource-limited environments as empty niche space determines habitat availability [64]. Stress alters the dynamics of biotic interactions [65] and accentuates the benefit of traits such as early vegetative growth [66]. Some competitive traits, such as resource allocation to increased vertical growth in sunflower, that may provide an advantage for procuring establishment in disturbed habitats against other colonizing plant species have been actively selected against in cultivars, where in an agricultural environment their proximity to other individuals is artificially manipulated [67]. Nevertheless, we found some evidence that crop-derived alleles contribute to competitive growth, suggesting that they are likely to aid in the expansion of hybrid plants in wild environments.

Although leaves in the LW treatment were comparatively smaller, crop-like (i.e. larger) leaf size was favored by selection only in the LW environment. Reduction in leaf size is a common response to water stress as it may be necessary to decrease resource loss and achieve equilibrium for maximal potential growth in relation to available resources [68]. Leaf size reduction is also important to increase water use efficiency [69]; however, too great a reduction may limit the photosynthetic ability in *Helianthus* species [70]. In addition, smaller leaves with a reduced boundary layer will not lower leaf temperature and thus will not create a water use efficiency benefit [71]. Greater petiole length may influence the relative advantage of leaf size as longer petioles promote leaves to twist away from direct sunlight [71]. It is difficult to generalize our leaf size results, as water availability may not always predict the same differential selection [72]. Cultivar-derived alleles led to reduced or increased leaf size in the LW treatment depending on the locus. Future studies should attempt to separate direct effects of leaf size on tolerance of water stress from indirect effects due to its correlation with other traits.

Selection also reversed across treatments for leaf pressure potential. Positive selection for leaf pressure potential, as observed in the LW treatment, indicates that plants with a greater ability to move water through transpiration had greater fecundity. Unfortunately, through a combination of low heritability, greater influence of treatment than genotype, and no prior studies of this characteristic in crop-wild hybrids, we are unable to assign a crop-like attribute for leaf pressure potential. Given that cultivated individuals display a greater degree of wilting relative to wild individuals when exposed to water stress [27], cultivars would presumably display greater susceptibility to water stress, in that they would be less able than wild individuals to maintain leaf pressure potential as water availability is reduced.

While the present study included fewer RILs, our QTL mapping results are comparable with previous studies that utilized a greater sample size in the same population [33] [12] [13]. Small sample size in combination with low heritability in mapping populations can result in an upward bias in the variance explained by each QTL [73]. The detection of a small number of QTL of large effect thus cannot be used as the rationale for the simple genetic architecture of traits. Another consequence of small sample size is the potential for the detection of false positive QTL [74], but the generation of permutated significant LOD values is an accepted method to account for this [75]. Importantly, the additive effect of the crop-derived allele in our study was in the same direction as predicted from the selection analysis for approximately half the QTL (excluding water content and fecundity QTL) detected in the LW treatment, suggesting that the repeated detection of selection favoring crop-derived alleles may be advantageous under a broader range of natural conditions than otherwise thought [14] [15].

For many QTL, although the additive effect of the crop-derived allele was in the direction of selection, it conferred a more wild-like phenotype. For example, although decreased leaf size is a wild-like trait, the crop-derived allele had a negative additive effect for three QTL affecting this trait across both treatments. This result is not uncommon in QTL studies [76]. In previous studies of the same sunflower population, mixed effects of crop-derived alleles were found for several morphological characters [33] [12] [13]. In lettuce (*Lactua sativa* × *L. serriola*), the allelic directions were as expected for QTL for major effect but mixed for QTL of intermediate effect [7] [78] [79]. Consequently, failure to include genetic architecture methodology may present an incomplete consideration of crop allele introgression.

One caveat to all QTL studies using RILs is that they are limited to allelic variation between the two parent individuals. Although the wild individual in our study was collected from a semi-arid site, its drought tolerance is unknown and may not be representative of wild sunflowers in general. Nevertheless, several studies have now found that crop-derived traits and QTL are predicted for selection in a wild environment [12] [13] [79], supporting the hypothesis that the ability for hybrids to colonize new ecological habitats may have arisen from the migration of crop-derived alleles into wild populations [80]. That some alleles confer an advantage in both cultivated and wild environments indicates that adaptability to cultivated environments is present in the natural variation of some wild populations [16]. Selection for crop-derived traits and QTL in a water stressed environment is surprising given that selection for growth traits in domesticated sunflowers has led to a growth-drought tolerance trade-off [24], and that if plants display weedy characteristics, they tend to have more acute reactions to water and nutrient stress [81]. However, the intermediate levels of drought tolerance and root architecture observed in weedy sunflowers may reflect a hybrid origin [27], given the persistence of introgression from cultivated sunflowers. The possibility for greater fitness in hybrids with variable amounts of crop genome may arise from heterosis, linkage, and transgressive segregation [82]. It is important to note, as in this study, that increases in fecundity may not necessarily result in range expansion or increased invasiveness [83]. Despite this, one study observed greater colonization success as demonstrated by increased seedling emergence and survivorship, as well as earlier emergence in radish hybrids producing greater fecundity [84]. Selection for crop-derived traits and alleles in wild environments may contribute to range expansion and/or increased invasiveness of hybrid individuals but multigenerational studies are needed to more adequately predict the long-term consequences of crop-to-wild gene flow.

## Supporting Information

Table S1 **Character means (standard error) for the cultivar parent and hybrid populations.**
(DOC)

Table S2 **Bivariate genotypic correlations.**
(DOCX)

## References

[1] Ellstrand NC, Prentice HC, Hancock JF (1999) Gene flow and introgression from domesticated plants into their wild relatives. Annu Rev Ecol Syst 30: 539–563.

[2] Kim S-C, Rieseberg LH (1999) Genetic architecture of species differences in annual sunflowers: implications for adaptive trait introgression. Genetics 153: 965–977. 10511571 10.1093/genetics/153.2.965 PMC1460779

[3] Rieseberg LH, Kim S-C, Randell RA, Whitney KD, Gross BL, et al (2007) Hybridization and the colonization of novel habitats by annual sunflowers. Genetica 129: 149–165. 16955330 10.1007/s10709-006-9011-y PMC2442915

[4] Jorgensen RB, Andersen B, Snow AA, Hauser TP (1999) Ecological risks of growing genetically modified crops. Plant Biotech J 16: 69–71.

[5] Rieseberg LH, Burke JM (2001) The biological reality of species: gene flow, selection, and collective evolution. Taxon 50: 47–67.

[6] Morjan CL, Rieseberg LH (2004) How species evolve collectively: implications of gene flow and selection for the spread of advantageous alleles. Mol Ecol 13: 1341–1356. 15140081 10.1111/j.1365-294X.2004.02164.x PMC2600545

[7] Laughlin KD, Power AG, Snow AA, Spencer LJ (2009) Risk assessment of genetically engineered crops: fitness effects of virus-resistance transgenes in wild *Cucurbita pepo* . Ecol Appl 19: 1091–1101. 19688918 10.1890/08-0105.1

[8] Snow AA, Pilson D, Rieseberg LH, Paulsen MJ, Pleskac N, et al (2003) A *Bt* transgene reduces herbivory and enhances fecundity in wild sunflowers. Ecol Appl 13: 279–286.

[9] Yang X, Xia H, Wang W, Wang F, Su J, et al (2011) Transgenes for insect resistance reduce herbivory and enhance fecundity in advanced generations of crop-weed hybrids of rice. Evol Appl 4: 672–684. 25568014 10.1111/j.1752-4571.2011.00190.x PMC3352537

[10] Ellstrand NC (2003) Dangerous Liaisons? When Cultivated Plants Mate with Their Wild Relatives. Baltimore: The Johns Hopkins University Press.

[11] Chapman MA, Burke JM (2006) Letting the gene out of the bottle: the population genetics of genetically modified crops. New Phytol 170: 429–443. 16626466 10.1111/j.1469-8137.2006.01710.x

[12] Baack EJ, Sapir Y, Chapman MA, Burke JM, Rieseberg LH (2008) Selection on domestication traits and quantitative trait loci in crop-wild sunflower hybrids. Mol Ecol 17: 666–677. 18179437 10.1111/j.1365-294X.2007.03596.x

[13] Dechaine JM, Burger JC, Chapman MA, Seiler GJ, Brunick R, et al (2009) Fitness effects and genetic architecture of plant-herbivore interactions in sunflower crop-wild hybrids. New Phytol 184: 828–841. 19656303 10.1111/j.1469-8137.2009.02964.x

[14] Whitton J, Wolf DE, Arias DM, Snow AA, Rieseberg LH (1997) The persistence of cultivar alleles in wild populations of sunflowers five generations after hybridization. Theor Appl Genet 95: 33–40.

[15] Snow AA, Culley TM, Campbell LG, Sweeney PM, Hegde SG, et al (2010) Long-term persistence of crop alleles in weedy populations of wild radish (*Raphanus raphanistrum*). New Phytol 186: 537–548. 20122132 10.1111/j.1469-8137.2009.03172.x

[16] Kane NC, Rieseberg LH (2008) Genetics and evolution of weedy *Helianthus annuus* populations: adaptation of an agricultural weed. Mol Ecol 17: 384–394. 17725567 10.1111/j.1365-294X.2007.03467.x

[17] Mercer KL, Wyse DL, Shaw RG (2006) Effects of competition on the fitness of wild and crop-wild hybrid sunflower from a diversity of wild populations and crop lines. Evolution 60: 2044–2055. 17133861

[18] Mercer KL, Andow DA, Wyse DL, Shaw RG (2007) Stress and domestication traits increase the relative fitness of crop-wild hybrids in sunflower. Ecol Lett 10: 383–393. 17498137 10.1111/j.1461-0248.2007.01029.x

[19] Cummings CL, Alexander HM, Snow AA (1999) Increased pre-dispersal seed predation in sunflower crop-wild hybrids. Oecol 121: 330–338. 10.1007/s004420050936 28308321

[20] Alexander HM, Cummings CL, Kahn L, Snow AA (2001) Seed size variation and predation of seeds produced by wild and crop-wild sunflower. Am J Bot 88: 623–627. 11302847

[21] Burke JM, Rieseberg LH (2003) Fitness effects of transgenic disease resistance in sunflowers. Science, New Series 300: 1250. 10.1126/science.1084960 12764188

[22] Davies WJ, Zhang J, Yang J, Dodd IC (2010) Novel crop science to improve yield and resource use efficiency in water-limited agriculture. J Agr Sci 149: 123–131.

[23] Bartlett MK, Scoffoni C, Sack L (2012) The determinants of leaf turgor loss point and prediction of drought tolerance of species and biomes: a global meta-analysis. Ecol Lett 15: 393–405. 22435987 10.1111/j.1461-0248.2012.01751.x

[24] Mayrose M, Kane NC, Mayrose I, Dlugosch KM, Rieseberg LH (2011) Increased growth in sunflower correlates with reduced defences and altered gene expression in response to biotic and abiotic stress. Mol Ecol 20: 4683–4694. 21988641 10.1111/j.1365-294X.2011.05301.x

[25] Agele SO (2003) Sunflower responses to weather variations in rainy and dry, cropping seasons in a tropical raiforest zone. Inter J Biotron 32: 17–33.

[26] Turhan H, Baser I (2004) In vitro and In vivo water stress in sunflower (*Helianthus annuus* L.). Helia 27: 227–236.

[27] Koziol L, Rieseberg LH, Kane NC, Bever JD (2012) Reduced drought tolerance during domestication and the evolution of weediness results from tolerance-growth trade-offs. Evolution 66: 3803–3814. 23206138 10.1111/j.1558-5646.2012.01718.x PMC3515206

[28] Erickson DL, Fenster CB, Stenoien HK, Price D (2004) Quantitative trait locus analyses and the study of evolutionary process. Mol Ecol 13: 2505–2522. 15315666 10.1111/j.1365-294X.2004.02254.x

[29] Ash M (2012) Sunflowerseed. Available: http://www.ers.usda.gov/topics/crops/soybeans-oil-crops/sunflowerseed.aspx#.UVB2zb-Ogy7/. Accessed 2013 June 15.

[30] Burke JM, Gardner KA, Rieseberg LH (2002a) The potential for gene flow between cultivated and wild sunflower (*Helianthus annuus*) in the United States. Am J Bot 89: 1550–1552. 21665757 10.3732/ajb.89.9.1550

[31] Harter AV, Gardner KA, Falush D, Lentz DL, Bye RA, et al (2004) Origin of extant domesticated sunflowers in eastern North America. Nature 430: 201–205. 15241413 10.1038/nature02710

[32] Heiser CB (1951) The sunflower among North American Indians. P Am Philos Soc 95: 432–448.

[33] Burke JM, Tang S, Knapp SJ, Rieseberg LH (2002b) Genetic analysis of sunflower domestication. Genetics 161: 1257–1267. 12136028 10.1093/genetics/161.3.1257 PMC1462183

[34] Arias DM, Rieseberg LH (1994) Gene flow between cultivated and wild sunflowers. Theor Appl Genet 89: 655–660. 24178006 10.1007/BF00223700

[35] Linder CR, Taha I, Seiler GJ, Snow AA, Rieseberg LH (1998) Long-term introgression of crop genes into wild sunflower populations. Theor Appl Genet 96: 339–347. 24710869 10.1007/s001220050746

[36] Dechaine JM, Burger JC, Burke JM (2010) Ecological patterns and genetic analysis of post-dispersal seed predation in sunflower (*Helianthus annuus*) crop-wild hybrids. Mol Ecol 19: 3477–3488. 20637050 10.1111/j.1365-294X.2010.04740.x

[37] USDA (2012) Web Soil Survey. Available websoilsurvey.nrcs.usda.gov/app/. Accessed 2013 July 24.

[38] Schneiter AA, Miller JF (1981) Description of sunflower growth stages. Crop Sci 21: 901–903.

[39] R Core Team (2013) R: A language and environment for statistical computing. Vienna: R Foundation for Statistical Computing.

[40] Bates D, Maechler M, Bolker B (2012) lme4: Linear mixed-effects models using S4 classes. Available http://CRAN.R-project.org/package=lme4. Accessed 2013 February 17.

[41] Scheipl F, Greven S, Kuechenhoff H (2008) Size and power of tests for a zero random effect variance or polynomial regression in additive and linear mixed models. Comput Stat Data An 52: 3283–3299.

[42] Kuznetsova K, Brockhoff PB, Christensen RHB (2013) lmerTest: Tests for random and fixed effects for linear mixed effect models (lmer objects of lme4 package). Available http://CRAN.R-project.org/package=lmerTest. Accessed 2013 April 11.

[43] Lenth RV (2013) lsmeans: Least-squares means. Available: http://CRAN.R-project.org/package=lsmeans. Accessed 2013 March 23.

[44] Geyer CJ, Wagenius S, Shaw RG (2007) Aster models for life history analysis. Biometrika 94: 415–426.

[45] Shaw RG, Geyer CJ, Wagenius S, Hangelbroek HH, Etterson JR (2008) Unifying life history analyses for the inference of fitness and population growth. The Amer Nat 172: E35–E47. 18500940 10.1086/588063

[46] Shaw RG, Geyer CJ (2010) Inferring fitness landscapes. Evolution 64: 2510–2520. 20456492 10.1111/j.1558-5646.2010.01010.x

[47] Stanton-Geddes J, Shaw RG, Tiffin P (2012) Interactions between soil habitat and geographic range location affect plant fitness. PLoS One 7: e36015. 22615745 10.1371/journal.pone.0036015 PMC3355151

[48] Orr HA (2009) Fitness and its role in evolutionary genetics. Nature Rev Genet 10: 531–539. 19546856 10.1038/nrg2603 PMC2753274

[49] Geyer CJ (2008) The Aster Package Tutorial. Available: http://www.stat.umn.edu/geyer/aster/library/aster/doc/tutor.pdf/. Accessed 2014 June13.

[50] Neter J, Kutner M, Nachtsheim C, Wasserman W (1996) Applied Linear Statistical Models. Chicago: Irwin.

[51] Li JT, Yang J, Chen DC, Zhang XL, Tang ZS (2007) An optimized mini-preparation method to obtain high-quality genomic DNA from mature leaves of sunflower. Genet Mol Res 6: 1064–1071. 18273799

[52] Bachlava E, Taylor CA, Tang S, Bowers JE, Mandel JR, et al (2012) SNP discovery and development of a high-density genotyping array for sunflower. PLoS ONE 7: e29814. 22238659 10.1371/journal.pone.0029814 PMC3251610

[53] Bowers JE, Bachlava E, Brunick RL, Rieseberg LH, Knap SJ, et al (2012) Development of a 10,000 locus genetic map of the sunflower genome based on multiple crosses. G3 2: 721–729. 22870395 10.1534/g3.112.002659 PMC3385978

[54] Becker RA, Chambers JM, Wilks AR (1988) The New S Language. Wadsworth & Brooks/Cole.

[55] Barker MS, Kane NC, Matvienko M, Kozik A, Michelmore RW, et al (2008) Multiple paleopolyploidizations during the evolution of the compositae reveal parallel patterns of duplicate gene retention after millions of years. Mol Biol Evol 25: 2445–2455. 18728074 10.1093/molbev/msn187 PMC2727391

[56] Lander ES, Green P, Abrahamson J, Barlow A, Daly MJ, et al (1987) MAPMAKER: an interactive computer package for constructing primary genetic linkage maps of experimental and natural populations. Genomics 1: 174. 3692487 10.1016/0888-7543(87)90010-3

[57] Lincoln S, Daly M, Lander E (1992) Constructing genetic maps with MAPMAKER/EXP 3.0. Available: http://home.cc.umanitoba.ca/~psgendb/birchhomedir/doc/mapmaker/mapmaker.tutorial.pdf. Accessed 2014 June13..

[58] Chapman MA, Pashley CH, Wenzler J, Hvala J, Tang S, et al (2008) A genomic scan for selection reveals candidates for genes involved in the evolution of cultivated sunflower (Helianthus annuus). Plant Cell 20: 2931–2945. 19017747 10.1105/tpc.108.059808 PMC2613673

[59] Wang S, Basten CJ, Zeng ZB (2010) Windows QTL Cartographer v.2.5 ed. : NCSU Statistical Genetics.

[60] Churchill GA, Doerge RW (1994) Empirical threshold values for quantitative trait mapping. Genetics 138: 963–971. 7851788 10.1093/genetics/138.3.963 PMC1206241

[61] Jiang C, Zeng Z (1995) Multiple trait analysis of genetic mapping for quantitative trait loci. Genetics 140: 1111–1127. 7672582 10.1093/genetics/140.3.1111 PMC1206666

[62] Gentzbittel L, Vear F, Zhang Y-X, Berville A, Nicolas P (1995) Development of a consensus linkage RFLP map of cultivated sunflower (Helianthus annuus L.). Theor Appl Genet 90: 1079–1086. 24173066 10.1007/BF00222925

[63] Tang S, Yu J-K, Slabaugh MB, Shintani DK, Knapp SJ (2002) Simple sequence repeat map of the sunflower genome. Theor Appl Genet 105: 1124–1136. 12582890 10.1007/s00122-002-0989-y

[64] Funk JL, Cleland EE, Suding KN, Zavaleta ES (2008) Restoration through reassembly: plant traits and invasion resistance. Trends Ecol Evolut 23: 695–703. 10.1016/j.tree.2008.07.013 18951652

[65] Liancourt P, Callaway RM, Michalet R (2005) Stress tolerance and competitive-response ability determine the outcome of biotic interactions. Ecology 86: 1611–1618.

[66] Stanton ML, Roy BA, Thiede DA (2000) Evolution in stressful environments. I. phenotypic variability, phenotypic selection, and response to selection in five distinct environmental stresses. Evolution 54: 93–111. 10937187 10.1111/j.0014-3820.2000.tb00011.x

[67] Weinig C (2000) Differing selection in alternative competitive environments: shade-avoidance responses and germination timing. Evolution 54: 124–136. 10937189 10.1111/j.0014-3820.2000.tb00013.x

[68] Chapin FS, Bloom AJ, Field CB, Waring RH (1987) Plant responses to multiple environmental factors. BioScience 37: 49–57.

[69] Nobel PS (1999) Physiochemical and environmental plant physiology. New York: Academic Press.

[70] Boyer JS (1982) Plant productivity and environment. Science 218: 443–448. 17808529 10.1126/science.218.4571.443

[71] Donovan LA, Dudley SA, Rosenthal DM, Ludwig F (2007) Phenotypic selection on leaf water use efficiency and related ecophysiological traits for natural populations of desert sunflowers. Oecol 152: 13–25. 10.1007/s00442-006-0627-5 17165094

[72] Donovan LA, Ludwig F, Rosenthal DM, Rieseberg LH, Dudley SA (2009) Phenotypic selection on leaf ecophysiological traits in *Helianthus* . New Phytol 183: 868–879. 19552693 10.1111/j.1469-8137.2009.02916.x

[73] Beavis WD (1998) QTL analyses: power, precision and accuracy. In: Paterson AH, editor. Molecular Dissection of Complex Traits. Boca Raton: CRC Press. pp. 145–162.

[74] Slate J (2013) From Beavis to beak color: a simulation study to examine how much QTL mapping can reveal about the genetic architecture of quantitative traits. Evolution 67: 1251–1262. 23617906 10.1111/evo.12060

[75] Mauricio R (2001) Mapping quantitative trait loci in plants: uses and caveats for evolutionary biology. Nature Rev Genet 2: 370–381. 11331903 10.1038/35072085

[76] Ross-Ibarra J (2005) Quantitative trait loci and the study of plant domestication. Genetica 123: 197–204. 15881692 10.1007/s10709-004-2744-6

[77] Argyris J, Truco JM, Ochoa O, Knapp SJ, Still DW, et al (2005) Quantitative trait loci associated with seed and seeding traits in *Lactua* . Theor Appl Genet 111: 1365–1376. 16177902 10.1007/s00122-005-0066-4

[78] Zhang FZ, Wagstaff C, Rae AM, Sihota AK, Keevil CW, Rothwell SD, Clarkson GJJ, Michelmore RW, Truco MJ, Dixon MS, Taylor G (2007) QTL for shelf life in lettuce co-locate with those for leaf biophysical properties but not for leaf developmental traits. Journal of Experimental Botany 58: 1433–1449. 17347132 10.1093/jxb/erm006

[79] Hartman Y, Hooftman DAP, Schranz ME, van Tienderen PH (2013a) QTL analysis reveals the genetic architecture of domestication traits in Crisphead lettuce. Genet Resour Crop Ev 60: 1487–1500.

[80] Reagon M, Snow AA (2005) Cultivated *Helianthus annuus* (Asteraceae) volunteers as a genetic “bridge” to weedy sunflower populations in North America. Am J Bot 93: 127–133.

[81] He W-M, Thelen G, Ridenour W, Callaway R (2010) Is there a risk to living large? Large size correlates with reduced growth when stressed for knapweed populations. Biol Invasions 12: 3591–3598.

[82] Hartman Y, Uwimana B, Hooftman DAP, Schranz ME, van de Wiel CCM, et al (2013b) Genomic and environmental selection patterns in two distinct lettuce crop-wild hybrid crosses. Evol Appl 6: 569–584. 23789025 10.1111/eva.12043 PMC3684739

[83] Cummings CL, Alexander HM (2002) Population ecology of wild sunflowers: effects of seed density and post-dispersal vertebrate seed predators. Oecol 130: 274–280. 10.1007/s004420100806 28547151

[84] Hovick SM, Campbell LG, Snow AA, Whitney KD (2011) Hybridization alters early life-history traits and increases plant colonization success in a novel region. The Amer Nat 179: 192–203. 22218309 10.1086/663684

