# Supplementary material for: Selection on Crop-Derived Traits and QTL in Sunflower (Helianthus annuus) Crop-Wild Hybrids under Water Stress
Source: PLoS One. 2014 Jul 21;9(7):e102717. doi: 10.1371/journal.pone.0102717 (PMC4105569; doi:10.1371/journal.pone.0102717)
Supplement: Table S1 — Character means (standard error) for the cultivar parent and hybrid populations. (DOC) [file pone.0102717.s001.doc]

**Table S1** Character means (standard deviation) for the cultivar parent and hybrid populations

|  | **Cultivar parent** | | **RIL** | |
| --- | --- | --- | --- | --- |
|  | **CW** | **LW** | **CW** | **LW** |
| **Fecundity** | 250.00 (354.00) | 81.30 (42.90) | 364.80 (380.40) | 106.50 (116.80) |
| **Stem Diameter (mm)** | 7.52 (3.20) | 6.41 (1.99) | 8.23 (5.32) | 5.39 (2.18) |
| **Plant Height (cm)** | 57.06 (12.09) | 56.13 (12.65) | 57.45 (25.39) | 42.89 (14.20) |
| **Petiole Length (cm)** | 6.65 (2.60) | 4.65 (2.13) | 5.32 (3.59) | 3.57 (1.94) |
| **Leaf Size (cm^2^)** | 78.70 (79.2) | 46.10 (34.80) | 90.46 (103.63) | 36.93 (29.00) |
| **Branch Number** | 1.67 (1.12) | 0.25 (0.50) | 5.49 (4.72) | 2.41 (1.98) |
| **Head Diameter (mm)** | 64.70 (26.50) | 59.20 (23.50) | 43.57 (15.74) | 35.52 (11.48) |
| **Head Total** | 2.25 (1.50) | 1.25 (0.50) | 10.69 (9.79) | 5.42 (2.92) |
| **Days to Flower** | 81.00 (4.90) | 82.50 (2.65) | 82.59 (13.07) | 86.64 (12.43) |
| **Water Content** | 25.80 (11.02) | 18.85 (0.79) | 21.05 (10.87) | 21.38 (7.21) |
| **Leaf Pressure Potential (MPa)** | 4.20 (1.10) | 4.03 (0.73) | 3.22 (1.40) | 4.40 (0.92) |

Morphological, reproductive, and physiological characters measured in cultivated sunflower [cmsHA89 (N=9)], as well as cultivar (cmsHA89) x wild (ann1238) recombinant inbred lines [RILs(N=237)], exposed to control water (CW) and low water (LW) treatment
