# Supplementary material for: Selection on Crop-Derived Traits and QTL in Sunflower (Helianthus annuus) Crop-Wild Hybrids under Water Stress
Source: PLoS One. 2014 Jul 21;9(7):e102717. doi: 10.1371/journal.pone.0102717 (PMC4105569; doi:10.1371/journal.pone.0102717)
Supplement: Table S2 — Bivariate genotypic correlations. (DOCX) [file pone.0102717.s002.docx]

**Table S2** Bivariate genotypic correlations

|  | **Fecundity** | **Stem**  **Diameter** | **Plant**  **Height** | **Petiole Length** | **Leaf**  **Size** | **Branch Number** | **Head**  **Total** | **Head**  **Diameter** | **Days to**  **Flower** | **Leaf PP** | **Water**  **Content** |
| --- | --- | --- | --- | --- | --- | --- | --- | --- | --- | --- | --- |
| **Fecundity** |  | 0.37 | 0.36 | 0.46 | 0.30 | **0.60** | **0.60** | 0.21 | 0.21 | -0.26 | -0.08 |
| **Stem**  **Diameter** | 0.32 |  | **0.92** | **0.88** | **0.96** | **0.82** | **0.84** | **0.78** | **0.80** | -0.13 | -0.20 |
| **Plant**  **Height** | 0.37 | **0.79** |  | **0.84** | **0.89** | **0.80** | **0.88** | **0.83** | **0.74** | -0.04 | -0.13 |
| **Petiole**  **Length** | 0.19 | **0.84** | **0.76** |  | **0.92** | **0.88** | **0.83** | **0.70** | **0.77** | -0.15 | -0.19 |
| **Leaf**  **Size** | 0.41 | **0.76** | **0.74** | **0.75** |  | **0.81** | **0.79** | **0.75** | **0.82** | -0.11 | -0.25 |
| **Branch Number** | 0.36 | **0.58** | 0.31 | 0.49 | 0.45 |  | **0.92** | **0.58** | **0.73** | -0.21 | -0.21 |
| **Head**  **Total** | 0.34 | **0.65** | 0.42 | 0.54 | 0.44 | **0.82** |  | **0.65** | **0.65** | -0.20 | -0.16 |
| **Head**  **Diameter** | **0.56** | **0.68** | **0.81** | **0.68** | **0.77** | 0.28 | 0.31 |  | **0.68** | 0.02 | -0.11 |
| **Days to**  **Flower** | 0.11 | 0.37 | **0.48** | 0.26 | 0.22 | 0.05 | 0.07 | 0.42 |  | -0.19 | -0.25 |
| **Leaf**  **PP** | -0.02 | 0.01 | 0.04 | 0.08 | -0.07 | -0.18 | -0.09 | -0.03 | 0.09 |  | 0.00 |
| **Water**  **Content** | -0.07 | 0.11 | -0.03 | 0.13 | -0.03 | 0.23 | 0.14 | -0.11 | -0.38 | 0.15 |  |

Bivariate genotypic correlations measured in sunflower cultivar (cmsHA89) x wild (ann1238) hybrids recombinant inbred lines (RILs) exposed to two watering treatments. Pearson correlation coefficients for the control and low water treatments are shown in the upper and lower portions, respectively. Significant relationships (*P* < 0.05) are bolded after adjustment using the Holm-Bonferroni method.
